# What’s new in the assessment of lupus activity?

**DOI:** 10.1093/rap/rkaf062

**Published:** 2025-05-26

**Authors:** Ana Isabel Ramos-Lisbona, Nur Azizah Allameen, David A Isenberg

**Affiliations:** Department of Rheumatology, Hospital General Universitario Gregorio Marañón, Madrid, Spain; Rheumatology Service, Department of Medicine, Woodlands Health, Singapore; Division of Medicine, Department of Ageing, Rheumatology and Regenerative Medicine, University College London, London, UK

**Keywords:** systemic lupus erythematosus, activity measurement, SLEDAI-2K, BILAG-2004, SLE-DAS, Easy-BILAG, composite indices, validation, comprehensive assessment

## Abstract

Capturing disease activity in SLE remains challenging. The binary nature of global score indices such as the SLEDAI 2000 (SLEDAI-2K) poses limitations, while the complexity of the BILAG-2004 index requires training and more time investment. Recent efforts to improve SLE activity indices include the SLE Disease Activity Score (SLE-DAS) and Easy-BILAG system. This review analyses the main indices used to assess SLE activity, examines their progressive refinements, evaluates their advantages and limitations and aims to identify the optimal index. The SLE-DAS offers greater sensitivity than the SLEDAI-2K and the Easy-BILAG simplifies scoring while maintaining the comprehensiveness of the BILAG-2004. Composite indices like the SLE Responder Index and BILAG-based Composite Lupus Assessment integrate the SLEDAI-2K and BILAG-2004 but are mainly used in clinical trials due to their complexity. This review emphasizes the importance of balancing sensitivity, specificity, simplicity and comprehensiveness in lupus activity measurement. The search for the optimal index remains ongoing.

Key messages

- Major efforts have been made to enhance the precision and reliability of evaluating lupus activity, especially in research settings.
- The SLE-DAS stands out for its practicality and efficiency, while the Easy-BILAG offers a more comprehensive approach with superior capacity to detect nuanced changes in disease activity.
- Despite significant advancements, no single lupus activity index is perfect, and the search for the ideal assessment tool continues.

## Introduction

Through the mid-1980s, approximately 60 attempts were made to define lupus activity using global scoring systems. These systems awarded points for clinical or serological features but lacked validation, reproducibility and sensitivity to change, rendering them of limited practical value [1, 2].

In the next decade, more refined indices were introduced, including the SLEDAI, the European Consensus Lupus Activity Measurement (ECLAM) and the Systemic Lupus Activity Measure (SLAM) [3, 4]. These demonstrated improved methodological rigor, including validation efforts, but remained incomplete [3]. Thus the SLEDAI fails to capture gastrointestinal (GI) involvement, ocular manifestations and haemolytic anaemia and cannot differentiate partial improvement, worsening or stable disease [4].The BILAG index was developed as a more comprehensive alternative [5]. Subsequent refinements—BILAG-2004 and SLEDAI-2K—improved accuracy and applicability. Despite these advancements, both still present challenges in comprehensiveness, sensitivity and practicality. The SLEDAI-2K has remained widely adopted due to its simplicity and efficiency, while the BILAG-2004 requires significant training and time investment [3, 5, 6].

Recent advancements, notably the SLE Disease Activity Score (SLE-DAS) [7, 8] and Easy-BILAG [9, 10], aim to overcome these limitations, offering improved sensitivity and usability. A detailed comparative analysis of these indices is still lacking. This review provides a comprehensive evaluation of traditional and emerging lupus activity indices, assessing their strengths and limitations and guiding their optimal application in clinical and research settings.

The objectives of this review are to analyse the evolution of lupus activity indices from traditional tools (SLEDAI, BILAG) to recent advancements (SLE-DAS, Easy-BILAG), highlight their respective strengths and limitations and assist clinicians and researchers in selecting the most appropriate tool for specific clinical and research contexts.

### Problems with currently used systems

Before discussing the SLEDAI-2K, it is pertinent to describe its predecessor, the SLEDAI. Developed by the Toronto Lupus Clinic, the SLEDAI was designed as a global scoring system to assess lupus activity through 24 weighted descriptors covering clinical and laboratory domains [11]. The index assigns scores based on the presence or absence of specific manifestations within the previous 10 days (later 30 days), producing a cumulative score ranging up to 105. Although widely used, the SLEDAI has a binary scoring system that fails to distinguish partial improvements, persistent disease activity or worsening. It omitted manifestations such as GI and ophthalmological involvement. These limitations led to the development of the SLEDAI-2K, which retains the structure of the SLEDAI while introducing modifications to improve its clinical utility [3, 4, 11].

The SLEDAI-2K consists of 24 descriptors across nine organ systems, including six clinical domains (central nervous system, vascular, musculoskeletal, serosal, dermal and constitutional) and three laboratory domains (renal, immunological and haematological). Weighted scores (1, 2, 4 or 8) are assigned based on their clinical relevance, with the total score still ranging up to 105 [3, 11]. This simplicity, combined with minimal training requirements, has made the SLEDAI-2K popular in both clinical and research settings [2]. A modification introduced in the SLEDAI-2K was the inclusion of persistently active manifestations, such as rash, alopecia, mucosal lesions and proteinuria, previously excluded unless new or recurring. This update provides a more accurate reflection of disease activity. However, it still has limitations. Although it introduced ophthalmologic symptoms such as scleritis and episcleritis, it still omits GI involvement and lacks the sensitivity to detect subtle changes in disease activity, limiting its effectiveness [4, 12].

The BILAG-2004 provides a more detailed, organ-based assessment by capturing 97 items across nine organ systems, including constitutional, mucocutaneous, neuropsychiatric, GI and ophthalmic domains. Disease activity is categorized into levels A–E based on clinical and laboratory findings, providing a comprehensive view of SLE manifestations. Its complexity presents challenges. Scoring using the standard BILAG-2004 format can occasionally take a considerable amount of time. While the time needed for individual assessments varies depending on case complexity and clinician experience, the multistep process for converting clinical observations into activity grades demands additional training, limiting its use in busy clinical settings [5, 6, 9].

These limitations have driven the development of newer assessment tools, e.g. SLE-DAS and Easy-BILAG, which seek to balance comprehensiveness and practicality [7, 9].

### The ‘new kids on the block’

Researchers in Portugal developed the SLE-DAS as a more comprehensive global index compared with the SLEDAI-2K. Unlike its predecessor, which relies on a binary scoring system, the SLE-DAS incorporates 17 clinical and laboratory variables, including alopecia, systemic and mucocutaneous vasculitis, arthritis, cardiac and pulmonary involvement, generalized and localized skin rash, mucosal ulcers, myositis, neuropsychiatric involvement, serositis, haemolytic anaemia, leukopenia, thrombocytopenia, hypocomplementemia, elevated anti-DNA and proteinuria >500 mg/24 h. These variables are assigned a dichotomous numerical value based on their presence (1 = yes, 0 = no). By including continuous variables such as the inflamed joints count (from the 28 joints assessed in DAS), leucocyte and platelet counts and 24-h proteinuria, the SLE-DAS achieves greater sensitivity to subtle changes in disease activity. The final score is calculated as a weighted sum of these variables using an online tool that generates a numerical result, similar to the DAS-28 in RA. This approach enhances the detection of changes in disease activity over time but requires slightly more time to compute than the SLEDAI-2K [7, 8]. A free online calculator for the SLE-DAS is available (http://sle-das.eu/).

While the SLE-DAS provides a comprehensive assessment of overall disease activity, including flare detection, the SLEDAI-2K also captures flares, but with more limited sensitivity. To address this, other specialized tools have been developed. Notably, the classic Safety of Estrogen in Lupus Erythematosus National Assessment Flare Index (c-SELENA FI) and the revised SELENA FI (r-SELENA FI) enhance the identification of lupus flares with a focused approach. The c-SELENA FI is a modification of the SELENA-SLEDAI flare index that evaluates clinical manifestations of flares without requiring serological markers. In contrast, the r-SELENA FI includes both clinical and serological parameters and is proposed to improve sensitivity in detecting flares. Unlike the SLE-DAS, which measures global disease activity while capturing flares, and SLEDAI-2K, which uses a binary approach for flare assessment, these indices are dedicated to flare detection [13, 14].

Despite the improvements introduced by the SLE-DAS, several variants of the SLEDAI-2K have been developed to address specific clinical needs [2].

The Systemic Lupus Erythematosus Pregnancy Disease Activity Index (SLEPDAI), a modification of the SLEDAI designed for use in pregnant women, has not yet been validated. It aims to account for pregnancy-related physiological changes that could confound disease activity measurements using the standard SLEDAI. This adaptation attempts to avoid false-positive results caused by normal pregnancy-related symptoms that may overlap with lupus activity [15, 16]. A study evaluating the SLE-DAS in pregnant women found a high correlation with the SLEPDAI during the first trimester. Both indices were identified as independent predictors of flares in the second and third trimesters, with the SLE-DAS demonstrating slightly better performance [16]. The SLE-DAS could be suitable for monitoring disease activity in pregnant women, but further studies are needed. The SLEDAI-2K Glucocorticoid Index (SLEDAI-2KG) adds glucocorticoid dosage as an additional variable to improve disease activity assessment. This modification enables more accurate representation of disease severity, as changes in corticosteroid dosage correlate with symptom improvements or worsening. This version offers a more dynamic measure of treatment response than the SLEDAI-2K, which only captures clinical manifestations [17].The Systemic Lupus Erythematosus Responder Index 50 (SRI-50) was designed to overcome the limitations in the SLEDAI-2K in capturing partial improvements. The SRI-50 defines a response as a ≥50% improvement in individual disease manifestations, provided that no new manifestations emerge and no existing ones worsen. This index offers enhanced sensitivity in clinical trials by detecting meaningful partial responses, which is critical in evaluating treatment efficacy. However, the SRI-50 solely captures improvement and fails to detect worsening in clinical features, limiting its utility as a comprehensive activity measure. It remains primarily a research tool, with limited use in routine practice [18]. Lastly, the clinical SLEDAI-2K, a variant that excludes serological variables such as anti-dsDNA and complement levels, was developed to facilitate use in clinical practice, particularly in settings where laboratory results are not readily available. It retains utility in guiding treatment decisions, as treatment intensification based solely on serological activity is not typically recommended [12].

Both the SRI-50 and clinical SLEDAI-2K illustrate that modifications to the SLEDAI-2K, while addressing specific gaps, retain its core structure focused on simplicity, but at the expense of comprehensiveness [2, 19]. Carter *et al.* [9] introduced the Easy-BILAG, a simplified version of the BILAG-2004 that reduces scoring complexity while maintaining accuracy. It was developed to identify key barriers to its use in daily practice and clinical trials, including scoring complexity, glossary accessibility and time constraints.

The Easy-BILAG retains the structure of the BILAG-2004 but incorporates modifications aimed at improving efficiency and usability. All descriptors from the BILAG-2004 remain, with rearranged items prioritizing frequently scored manifestations and a colour-coded scoring system that enhance its usability. The Easy-BILAG reorganizes the layout based on data from the BILAG Biologics Register (BILAG-BR). The most frequent manifestations are listed on the first page, with less frequent ones relegated to a second page, which requires assessment only if indicated, facilitated by screening questions [9, 10].

Another modification is the integration of clinical definitions. In the BILAG-2004, these definitions are in a separate glossary, while the Easy-BILAG incorporates concise definitions adjacent to each clinical variable, improving readability and adherence to scoring criteria [9].

The scoring system for assigning domains A–E, previously dependent on complex algorithms described in a separate document, is simplified. The Easy-BILAG integrates instructions within the scoring form and uses colour-coding to facilitate the assignment of activity levels (grades A–E), reducing errors and completion time [9]. Easy-BILAG and its training materials are freely available at https://licensing.leeds.ac.uk/products/healthcare-questionnaires.

The pros and cons of the new indices are shown in Table 1.

**Table 1.** Summary of the pros and cons of each lupus activity index, highlighting their distinct strengths and complementary roles in clinical and research settings

Criterion	SLE-DAS	SLEDAI-2K	BILAG-2004	Easy-BILAG	LLDAS
Discriminatory ability	Superior for flares and subtle changes (T2T)	Limited (binary scoring)	Comprehensive (organ based)	Comprehensive (organ-based, simplified format of the BILAG-2004)	Limited (binary; low disease state)
Granularity of disease assessment	Captures global activity but omits some organ-level details (e.g. urinary sediment)	Limited granularity (focuses on severe events) and does not reflect partial improvement	Very detailed assessment by organ/system	Same as the BILAG-2004 (retains its structure)	Binary definition; reflects overall disease control
Reliability and reproducibility	High (validated cut-offs)	High (validated; consistent results in routine practice)	High (validated, requires training for consistent scoring)	High (validated; improves interobserver agreement)	High (validated definition; used inT2T studies)
Ease of use	Easy (3–5 min^a^, online calculator)	Very easy (3–5 min^a^)	Moderate to complex (3–15 min^a^)	Moderate (2–7 min^a^)	Relatively easy (requires multiple criteria)
Best suited for	T2T strategies, routine practice and clinical trials	Routine practice, quick assessments	Organ-level assessment in routine practice and clinical trials	Organ-level assessment in routine practice and clinical trials	Defining low disease activity and T2T endpoints

a The estimated completion times for the BILAG-2004 and Easy-BILAG are based on Carter *et al.* [9] and clinical experience, as scoring time varies significantly with case complexity and clinician expertise. Similarly, since no specific studies have assessed the time required to complete the SLE-DAS and SLEDAI-2K, these estimates are approximations derived from expert opinion and are likewise influenced by case complexity and clinician expertise. Additionally, all these indices require laboratory data (e.g. haematology, renal and immunology parameters), which may not be immediately available in the clinic and must be added later, potentially extending the total time needed for a complete assessment.

The Lupus Low Disease Activity State (LLDAS) is widely adopted in the Asia–Pacific region. Unlike the SLEDAI-2K and BILAG-2004, which capture disease activity at any given time, the LLDAS provides a framework for defining a controlled disease state, offering clinicians a standardized treatment target. A comparative analysis between the SLE-DAS, Easy-BILAG and LLDAS is addressed in later sections [20].

## Discussion

The complexity and potential severity of SLE necessitates the development of a high-quality activity index. Such an index must integrate flare detection as a fundamental aspect of disease activity monitoring, enabling timely identification of exacerbations to prevent cumulative damage [21]. By encompassing flare detection within a broader framework of disease monitoring, the index should provide precise guidance on treatment adjustments, including the feasibility of medication reduction, to minimize adverse effects while maintaining disease control [22].

To meet these objectives effectively, an SLE activity index should possess several key attributes. It should be sensitive enough to detect even subtle changes in disease activity, which may hold significance for patients. It should demonstrate specificity in distinguishing between disease activity and other factors influencing symptoms, such as comorbidities and treatment side effects. The index should be user friendly, without imposing an unmanageable administrative burden. Its clinical relevance is paramount. It should accurately reflect disease activity, be validated in clinical studies to demonstrate its reliability and show consistent results across various populations. Lastly, the index should be adaptable to accommodate the diverse clinical manifestations of SLE patients [7, 23, 24].

### Comparative analysis of the SLE-DAS and SLEDAI-2K

The SLE-DAS improves the limitations of the SLEDAI-2K by incorporating continuous variables, allowing better discrimination of subtle changes in disease activity. This enables the definition of precise cut-off points for remission and different activity states, facilitating its implementation in treat-to-target (T2T) strategies [7, 8, 24, 25].

A key limitation of the SLEDAI-2K is its binary scoring system, which does not distinguish partial improvements or worsening of symptoms. In contrast, the SLE-DAS assigns weighted values to arthritis, proteinuria, leukopenia and thrombocytopenia, providing a more dynamic assessment. While the SLEDAI-2K applies a fixed weighting system prioritizing severe manifestations such as vasculitis over mucocutaneous symptoms, it does not distinguish between localized and generalized rash, assigning the same score regardless of its extent. This lack of granularity leads to underestimation of clinically relevant dermatologic activity. In comparison, the SLE-DAS captures a broader spectrum of disease activity, including cardiopulmonary and GI involvement, which the SLEDAI-2K omits. These differences enhance its sensitivity in routine monitoring and clinical trials [7, 8, 23, 25].

In terms of predictive value, the SLE-DAS has superior accuracy in detecting clinically significant changes in disease activity compared with the SLEDAI-2K. A study showed that a change in the SLE-DAS ≥1.72 indicated clinically significant worsening with a sensitivity of 95.5% and a specificity of 98.2%, while a similar change reflected improvement with a sensitivity of 89.5% and a specificity of 100%. The SLE-DAS has shown superior predictive value for long-term damage accrual compared with the SLEDAI-2K, which is relevant in clinical trials where sensitivity to change is critical [7, 8, 25].

Flare detection is another area where the SLE-DAS outperforms the SLEDAI-2K. The SLE-DAS demonstrated high accuracy in detecting and classifying flares, with a sensitivity of 97.1% and a specificity of 97.3% [8, 26]. The limited sensitivity of the SLEDAI-2K in detecting improvement may explain why certain therapies, such as rituximab, failed to demonstrate efficacy in clinical trials [8, 26].

The SLE-DAS has also been validated for flare detection. Validation criteria for flare definition according to the SLE-DAS were assessed and compared with the SLEDAI-2K, c-SELENA FI and r-SELENA FI. The SLE-DAS demonstrated high accuracy in detecting flares and classifying their severity, underscoring its potential for T2T strategies in routine clinical practice [8, 26].

While the c-SELENA FI and r-SELENA FI were also accurate in detecting flares, their sole function is flare detection, making them less versatile than the SLE-DAS, which measures overall disease activity and flare occurrence. The SLEDAI-2K showed inferior sensitivity for flare detection compared with the SLE-DAS [8].

The c-SELENA FI and r-SELENA FI are tools designed for flare detection and their application is time-consuming, unlike the SLE-DAS. This constitutes another advantage of the SLE-DAS, facilitating its use for decision-making in routine clinical practice and highlighting its superiority over previous global indices [7, 23].

Despite these strengths, some limitations of the SLE-DAS must be recognized. Most study populations have been Caucasian adults from Europe, making it uncertain whether the index applies to different environments and ethnicities. The classification of flare severity based on the degree of activity measured by the SLE-DAS requires further validation studies and currently does not distinguish between moderate and severe flares (it classifies them into mild or moderate/severe). In order to complete it, laboratory results are necessary, which are rarely available prior to the clinical visit [8, 25, 27–29].

While the SLE-DAS offers superior sensitivity through continuous variables, the SLEDAI-2K remains valuable for its simplicity and familiarity. The SLE-DAS may be better suited for research and T2T strategies, as it is more sensitive to subtle changes in disease activity and provides validated thresholds for disease states, making it particularly effective for assessing therapeutic responses in clinical trials. In contrast, the SLEDAI-2K retains practicality in settings where rapid, scoring without additional tools is prioritized.

### Comparative analysis of the Easy-BILAG and BILAG-2004

In contrast to SLE-DAS, which is an evolution from the SLEDAI-2K, the Easy-BILAG is a simplified version of the BILAG-2004. While the BILAG-2004 remains the most comprehensive tool for assessing organ-specific lupus activity, its use in routine clinical practice remains limited. The Easy-BILAG streamlines the BILAG-2004 scoring system, preserving the sensitivity and comprehensiveness of its predecessor. It improves efficiency by reducing scoring time, while remaining the only index designed to evaluate changes both globally and within individual organ domains. The Easy-BILAG reduced scoring time (median 59.5 min *vs* 80 min; *P* = 0.04) and improved interrater agreement. These times correspond to a validation exercise with 10 case vignettes, not individual patient assessments [5, 6, 9, 10, 25].

Although the BILAG-2004 has been reported to take an average of ≈8 min per case in clinical settings [5], scoring times vary considerably depending on case complexity and user experience. During clinical practice, we have observed that experienced users of the BILAG-2004 regularly complete assessments in <5 min, reflecting the impact of familiarity and long-term expertise with the index. This suggests that while the Easy-BILAG offers time savings, the efficiency gap between the two indices may be narrower for experienced clinicians. For those less familiar with the BILAG-2004, the Easy-BILAG is a more accessible alternative without compromising comprehensiveness.

### Comparative analysis of the SLE-DAS and Easy-BILAG

The SLE-DAS and Easy-BILAG adopt distinct approaches to assessing disease activity. No study has directly compared their sensitivity and direct comparative studies are needed to facilitate future research [7–9, 25, 30].

Both the SLE-DAS and Easy-BILAG have been validated. However, their application in clinical practice differs. The SLE-DAS, despite its advantages, has had limited use in real-world settings. Published studies have primarily applied it to Caucasian European populations, although research in other populations is emerging [27, 28, 31]. In contrast, the Easy-BILAG is a variant of an index in use for >30 years, extensively validated and has been employed in numerous clinical trials [8, 25, 32].

Beyond their validation, these indices also differ in their scoring methodology and in their coverage of clinical and laboratory parameters. The SLE-DAS, despite providing a more comprehensive assessment than the SLEDAI-2K, overlooks several manifestations commonly associated with severe disease activity, including constitutional symptoms, lymphadenopathy, splenomegaly, ocular symptoms and lupus pancreatitis. In contrast, the BILAG captures these manifestations through its organ-based domains [5, 25, 30].

The distinction between the SLE-DAS and BILAG is particularly notable in renal assessment. The BILAG evaluates blood pressure, glomerular filtration rate, serum creatinine, recent renal biopsy findings and active urinary sediment, making it the most comprehensive tool for detecting renal involvement, whereas the SLE-DAS relies solely on proteinuria to assess renal disease activity [5, 7, 9, 25]. While proteinuria is a key marker of lupus nephritis, relying solely on it to assess renal disease activity is concerning. Persistent proteinuria can reflect chronic damage rather than active inflammation, which highlights the importance of proper attribution. In both the SLEDAI and SLE-DAS, descriptors such as proteinuria should only be scored when attributable to active disease, as determined by clinical judgment and supporting evidence. Conversely, active lupus nephritis may occur with minimal proteinuria but significant urinary sediment abnormalities, such as haematuria or cellular casts, which the SLE-DAS does not include [23]. The developers of the SLE-DAS emphasize that proteinuria, treated as a continuous variable, avoids the dichotomous bias of the SLEDAI-2K and is the most sensitive sign of nephritis. They opted to exclude urinary sediment due to its operator-dependent variability and non-specific nature, as leukocyturia and haematuria may arise from infections, menstruation or kidney stones [29]. While this rationale is valid, standardized interpretation of sediment could enhance renal assessment and mitigate this limitation, making it a valuable addition to lupus nephritis monitoring.

The SLE-DAS and Easy-BILAG also differ significantly in efficiency and practical application. Although no studies have evaluated the exact time required to calculate the SLE-DAS, it is generally completed within a very short time using an online calculator. This, along with the absence of a need for specialized training, makes it particularly advantageous in settings where time for patient assessment is very limited. In contrast, the Easy-BILAG, although faster than the BILAG-2004, still requires more time than the SLE-DAS but offers a more detailed, organ-based assessment. This difference in efficiency may influence their applicability in different contexts. The SLE-DAS may be better suited for routine monitoring and T2T strategies, while the Easy-BILAG is probably more valuable for clinical trials and cases requiring comprehensive organ-specific monitoring. Together, these indices complement each other, with the SLE-DAS serving as a rapid tool for global assessment and the Easy-BILAG providing detailed, organ-specific monitoring [7, 9, 10, 25].

Both indices represent notable advancements over their predecessors. We acknowledge the strengths of the SLE-DAS, but its renal assessment remains a limitation. The Easy-BILAG improves upon the complexity of the BILAG-2004, yet its main advantage lies in facilitating scoring for less-experienced users. In contrast, the BILAG remains the most comprehensive tool for organ-specific assessment, particularly in complex cases. Both the SLE-DAS and BILAG are accessible and sensitive to change, but they excel in different areas: the SLE-DAS is well-suited for global activity monitoring and T2T strategies, while the BILAG offers superior organ-specific detail.

### LLDAS: definition, measurement and comparison with the SLE-DAS and Easy-BILAG

The LLDAS represents a T2T approach in the management of SLE. Unlike global activity indices such as the SLE-DAS and Easy-BILAG, which measure disease activity at a single point in time, the LLDAS defines a stable, low-activity state associated with reduced flares, less organ damage accrual and better long-term outcomes. The LLDAS was developed as a composite outcome measure to guide therapeutic decisions and has been increasingly used in clinical trials as a primary endpoint. Its validation across multiple international cohorts has demonstrated that patients achieving and maintaining the LLDAS experience had significantly improved survival rates and reduced morbidity [20, 33, 34].

The LLDAS is defined by meeting all five of the following criteria simultaneously: SLEDAI-2K ≤4, with no activity in major organ systems (renal, central nervous system, cardiopulmonary) and no haemolytic anaemia or GI activity; no new lupus disease activity compared with the previous assessment; Physician Global Assessment (PGA) ≤1 on a scale of 0–3; a current prednisone (or equivalent) dose of ≤7.5 mg/day and no use of prohibited immunosuppressants or biologics outside standard treatment protocols [20, 33, 34].

Measurement of the LLDAS is straightforward and relies on commonly used clinical parameters such as the SLEDAI-2K and PGA, making it practical for routine clinical application. Unlike the continuous scoring systems of the SLE-DAS and Easy-BILAG, the LLDAS is a binary measure (present or absent) that defines a stable, low-activity state based on predefined clinical and treatment criteria. This binary approach lacks sensitivity to subtle changes but excels as a therapeutic target, offering clinicians a standardized goal for patient management [9, 20, 33, 34].

Thus the LLDAS focuses on disease control rather than disease activity, distinguishing it from the SLE-DAS and Easy-BILAG. Its widespread use as a T2T endpoint underscores its importance in therapeutic strategies. Together, these indices address complementary aspects of lupus management: the SLE-DAS and Easy-BILAG as measures of activity and the LLDAS as a measure of disease control [20, 25, 33, 34].

### Composite indices for assessing treatment response: the Systemic Lupus Erythematosus Responder Index (SRI) and the BILAG-based Combined Lupus Assessment (BICLA) 

In an attempt to maximize the detection of response to new treatments by incorporating the strengths of both the SLEDAI and BILAG, composite indices have been developed, namely the SRI and BICLA. These indices are primarily used to define response in clinical trials. According to the SRI 4, a response requires an improvement in the SLEDAI score by at least 4 points, with no worsening in the BILAG or the PGA beyond a defined threshold. Similarly, for the BICLA, response is determined by an improvement in the BILAG score (all As and Bs must improve), while allowing for a limited degree of worsening in the SLEDAI and PGA under specific conditions [18, 35, 36].

The PGA is a subjective yet integral component of these composite indices. It is typically assessed on a visual analogue scale (VAS) ranging from 0 (no disease activity) to 3 or 10 (maximum disease activity), incorporating clinical manifestations, laboratory results and the physician’s overall judgment. By complementing objective indices such as the SLEDAI and BILAG, the PGA provides additional nuance to response assessment. However, despite their robustness, composite indices like the SRI and BICLA are specifically designed for clinical trials and not practical for routine clinical care due to the complexity of their application [18, 35, 36].

To synthesize the comparison of lupus activity indices discussed in this review, Table 2 provides an overview of their applicability across different clinical and research contexts. It highlights the suitability of each measurement for clinical trials, routine practice and specific manifestations of lupus activity.

**Table 2.** Applicability of lupus activity indices in clinical and research contexts

Category	Suitable measurements
Clinical trials	SRI, BICLA (composite indices designed for clinical trials), SLE-DAS (offers high sensitivity and specificity), Easy-BILAG (simplified for comprehensiveness), LLDAS (useful as a T2T goal and widely used as an endpoint in clinical trials)
Daily clinical practice	SLE-DAS (practical, fast and validated tool), Easy-BILAG (simplified scoring process), clinical SLEDAI-2K (eliminates serological variables for easier application), LLDAS (marker of disease control and validated therapeutic target)
Activity of cutaneous lupus	SLE-DAS (includes cutaneous activity markers like rash), Easy-BILAG (comprehensive across organ systems)
Activity of SLE (mucocutaneous and/or musculoskeletal)	SLE-DAS (mucocutaneous, musculoskeletal involvement covered), Easy-BILAG (comprehensive scoring for systemic activity), SLEDAI-2K (commonly used)
Activity of lupus nephritis (class III/IV)	BILAG (includes detailed renal parameters), Easy-BILAG (simplified renal assessment), SLE-DAS (relies on proteinuria, but limited specificity to distinguish damage from active disease)
Other organ involvement in SLE	BILAG and Easy-BILAG (comprehensive for systems like neuropsychiatric, GI and haematological involvement), SLE-DAS (includes additional parameters like cardiopulmonary and systemic vasculitis involvement)

Data sourced from Jesus *et al*. [7], Carter *et al*. [9], Franklyn *et al.* [20] and Inês *et al.* [25].

Global disease activity indices are a valuable tool for monitoring SLE activity, evaluating multiple systems simultaneously and providing a score that allows comparisons between different patients and within the same patient over time. The SLE-DAS is distinguished for its practicality, whereas the Easy-BILAG can capture activity more comprehensively and has the capacity to detect changes both globally and within each of its categories. Neither is completely devoid of drawbacks. Treatment change still requires physician input. The development of a perfect index may indeed be a chimera, but attempts to improve on the currently most widely used indices—the SLEDAI-2K and BILAG-2004—have gotten us closer.

Emerging tools such as the Cutaneous Lupus Erythematosus Disease Area and Severity Index, currently under qualification review by the US Food and Drug Administration for use in skin assessment, the LAMDA score for musculoskeletal involvement and the Treatment Response Measure for SLE Clinical Trials represent promising developments.

## Data Availability

The data underlying this article will be shared upon reasonable request to the corresponding author.
